# Molecular profiling of circulating tumor cells guides effective EGFR inhibitor treatment in advanced hepatocellular carcinoma: a case report

**DOI:** 10.3389/fonc.2025.1608604

**Published:** 2025-09-17

**Authors:** Wei Chiu, San-Chi Chen, Yee Chao, Jan‐Mou Lee

**Affiliations:** ^1^ Department of Medical Education, Taipei Veterans General Hospital, Taipei, Taiwan; ^2^ Department of Oncology, Taipei Veterans General Hospital, Taipei, Taiwan; ^3^ Faculty of Medicine, National Yang Ming Chiao Tung University, Taipei, Taiwan; ^4^ Department of Medicine, Central Clinic and Hospital, Taipei, Taiwan; ^5^ FullHope Biomedical Co., Ltd, New Taipei City, Taiwan

**Keywords:** hepatocellular carcinoma, circulating tumor cells, EGFR inhibitor, case report, precision diagnosis

## Abstract

Circulating tumor cells (CTCs) hold promise for use in personalized medicine for hepatocellular carcinoma (HCC). Their abundance and molecular characteristics may predict disease prognosis and could be used to monitor responses to treatment in specific types of cancers. The present case report described a 52-year-old woman with metastatic HCC with an extensive treatment history. The patient’s CTCs demonstrated EGFR surface mutations, specifically, an EGFR exon 19 deletion and an L858R mutation. Based on this finding, the patient’s metastatic lung lesions were treated with an EGFR tyrosine kinase inhibitor-based regimen, which achieved a partial response that was confirmed using the Response Evaluation Criteria in Solid Tumors (version 1.1). Notably, the patient benefited from this therapy regimen for a period of 14 months. The progression of precision medicine in HCC has been hampered by difficulties in identifying cancer driver genes and the limited utilization of histological diagnosis. The findings of the present study suggest that molecular analysis of CTCs may have the potential to guide personalized HCC treatment strategies in the future.

## 1 Introduction

Hepatocellular carcinoma (HCC) is one of the top three leading causes of cancer-related death worldwide (1). Systemic treatments for unresectable HCC include programmed cell death 1 (PD-1) blockade, cytotoxic T lymphocyte-associated protein-4 blockade, anti-VEGF antibodies and multiple-kinase inhibitors (2). However, the efficacy of these treatments as monotherapies is limited. Therefore, the identification of biomarkers to select individuals that may be more likely to respond to treatment is essential. HCC is often diagnosed using imaging techniques, due to the risk of bleeding and tumor cell seeding from tumor biopsies (3). This constraint prevents comprehensive research of the tumor biology and limits the development of biomarker-driven precision medicine for HCC (4).

Circulating tumor cells (CTCs) are cells shed from primary tumors, the study of which has potential for the development of precision medicine for cancer treatment (5–7). Analysis of the quantitative abundance, molecular characteristics and genomic heterogeneity of CTCs could be used predict disease prognosis and monitor response to therapy in patients with HCC (6). Li et al. (8) reported that patients with phosphorylated ERK+ (pERK)/pAkt- CTCs were more sensitive to Sorafenib, a multikinase inhibitor approved for the treatment of advanced HCC. They found that when the proportion of pERK^+^/pAkt^-^ expression in CTCs exceeded 40%, patients would experience longer progression-free survival compared to those with less than 40%. Furthermore, Winograd et al. (9) demonstrated that HCC patients with advanced stages have higher programmed death-ligand 1 (PD-L1)-expressing CTCs than patients with early stage. The authors also reported that the presence of PD-L1^+^ CTCs could predict the response to anti-PD-1 therapy. Additionally, recent GEO database-driven analyses have highlighted promising molecular targets in HCC, including hub genes identified through integrative differential expression, functional association, and protein–protein interaction studies (10), as well as CDT1 revealed by TCGA/GEO-based machine learning model in non-alcoholic fatty liver disease (NAFLD)-related HCC progression (11). Molecular analysis of CTCs expressing specific markers and therapeutic targets allow prediction of treatment response, thereby supporting the development of precision medicine in HCC (6, 12, 13).

The present study reported the case of a patient with advanced HCC with CTCs characterized by intracellular mutant EGFR, specifically containing an exon 19 deletion and L858R mutation.

## 2 Case presentation

**Figure 1:**
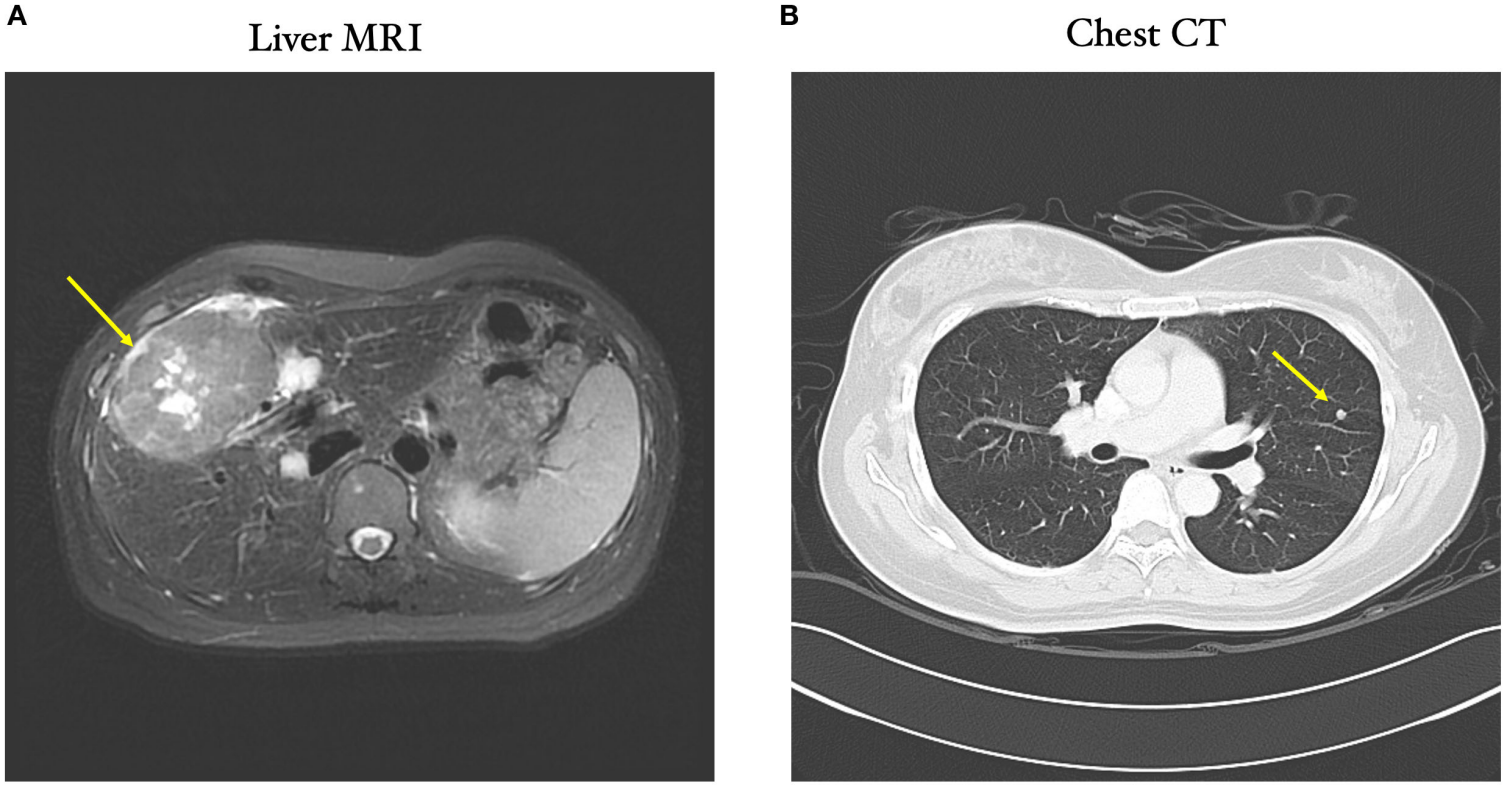
Liver MRI and CT scans at the time of the initial patient diagnosis. **(A)** MRI scan showing lobulated soft tissue mass predominantly at right lobe of liver, with mild high signal intensity on T2 weighted image (T2WI) (yellow arrow) and **(B)** CT scan showing nodular shadow at right upper lobe of lung (yellow arrow). A T2WI is a standard MRI sequence used for evaluating focal liver lesions. HCC typically shows higher signal intensity on T2WI compared to normal liver parenchyma.

A 52-year-old woman presented to Taipei Veterans General Hospital in May 2018 with intermittent right upper quadrant pain and abdominal fullness that had persisted for numerous months. MRI and chest CT scans demonstrated multiple liver tumors and lung metastases, respectively (**Figure 1**). The patient’s α-fetoprotein (AFP) levels were notably elevated (12,154 ng/ml) compared to normal AFP levels (0 to 40 ng/ml). She was positive for hepatitis B surface antigen with HBV viral load 284,000 IU/ml. The patient was subsequently diagnosed with stage IV hepatitis B-related HCC (tumor stage, T3aN0M1), classified as Barcelona Clinic Liver Cancer stage C.

The patient underwent a number of treatments between May 2018 and December 2021, which included Y^90^ radioembolization, transarterial chemoembolization, bevacizumb and atezolozumab, sorafenib, lenvatinib, the NBM-BMX clinical trial, fluorouracil and oxaliplatin chemotherapy regimen, pembrolizumab, cabozantinib and ramucirumab. A detailed timeline of systemic treatment was provided in **Supplementary Table 1**. However, all of the systemic therapies resulted in a best response of stable disease according to the Response Evaluation Criteria in Solid Tumors (version 1.1) (14). Given the refractory nature of the disease, CTC molecular investigation was carried out to provide more insight for choice of drug.

CTC evaluation was performed as follows: A total of 2.5 × 10^5^ peripheral blood mononuclear cells (PBMCs) were aliquoted, isolated from 125 μL of peripheral blood, and stained with antibodies specific for CTC identification and phenotype analysis. Cells were sequentially subjected to surface marker staining, cell membrane permeabilization, fixation and intracellular staining. Antibodies used in immunostaining were composed of the following CTC identification antibodies: CD45 (1:40; clone J33; cat. no. A07784; Beckman Coulter, Inc.), CD326/epithelial cell adhesion molecule (EpCAM; 1:40; clone 9C4; cat. no. 324236; BioLegend, Inc.), cytokeratin (CK-7/-8; 1:80; clone CAM5.2; cat. no. 347653; BD Pharmingen; BD Biosciences), cytokeratin-14, 15, 16, and 19 (1:160; clone KA1; cat. no. 563648; BD Pharmingen; BD Biosciences), human leukocyte antigen (HLA)-A, B and C (Major Histocompatibility Complex, Class I; 1:40; clone W6/32; cat. no. 311438; BioLegend, Inc.). Phenotype analysis antibodies used were as follows: EGFR E746-A750del specific (1:50; clone D6B6; cat. no. 54016S; CST Biological Reagents Co., Ltd.) and EGFR L858R mutant specific (1:50; clone 43B2; cat. no. 64716S; CST Biological Reagents Co., Ltd.). All stained cells were subsequently analyzed using a Gallios flow cytometer (Beckman Coulter, Inc.). All data were analyzed using the Kaluza software (Beckman Coulter, Inc.). Calibration of the flow cytometer was performed daily with Flow-Check pro beads (Beckman Coulter, Inc.) before each experiment. Compensation for each fluorescent signal was set using either identical samples stained with isotype controls or unstained samples.

CTCs were identified using a series of gating steps. Briefly, the EpCAM^+^ and high-SSC cells were gated. Secondly, a population with cytokeratin positivity was identified in the dot plot panel with cytokeratin against CK-14, -15, -16 and -19. Finally, cells expressing CD45^-^, HLA-A, B and C^+^ were gated (**Supplementary Figure 1**). The number of CTCs were determined from the gated population. CTCs expressing mutant EGFR (exon 19 deletion and L858R mutation) were identified and quantified as percentages of the total CTC population. CTCs evaluation according to the above methods were performed in following time points: Jan 2022, Mar 2022, Sep 2022 and Mar 2023. (**Supplementary Figure 2**, **Supplementary Table 2**).

Positive staining of surface EGFR exon 19 deletion was detected, with a percentage of 24.7, in the first CTCs investigation in Jan 2022 (**Supplementary Table 2**). As a result, EGFR TKI erlotinib was added to the treatment regimen. Over the next 2 months, a significant decrease in AFP levels (from 3,880–294 ng/dl) and CTC counts (from the initial 23 to 782 cells/2.5 × 10^5^ PBMCs) was observed (**Supplementary Table 2**), and a PR reported from a follow-up chest CT scan, according to the Response Evaluation Criteria in Solid Tumors (version 1.1).

**Figure 2:**
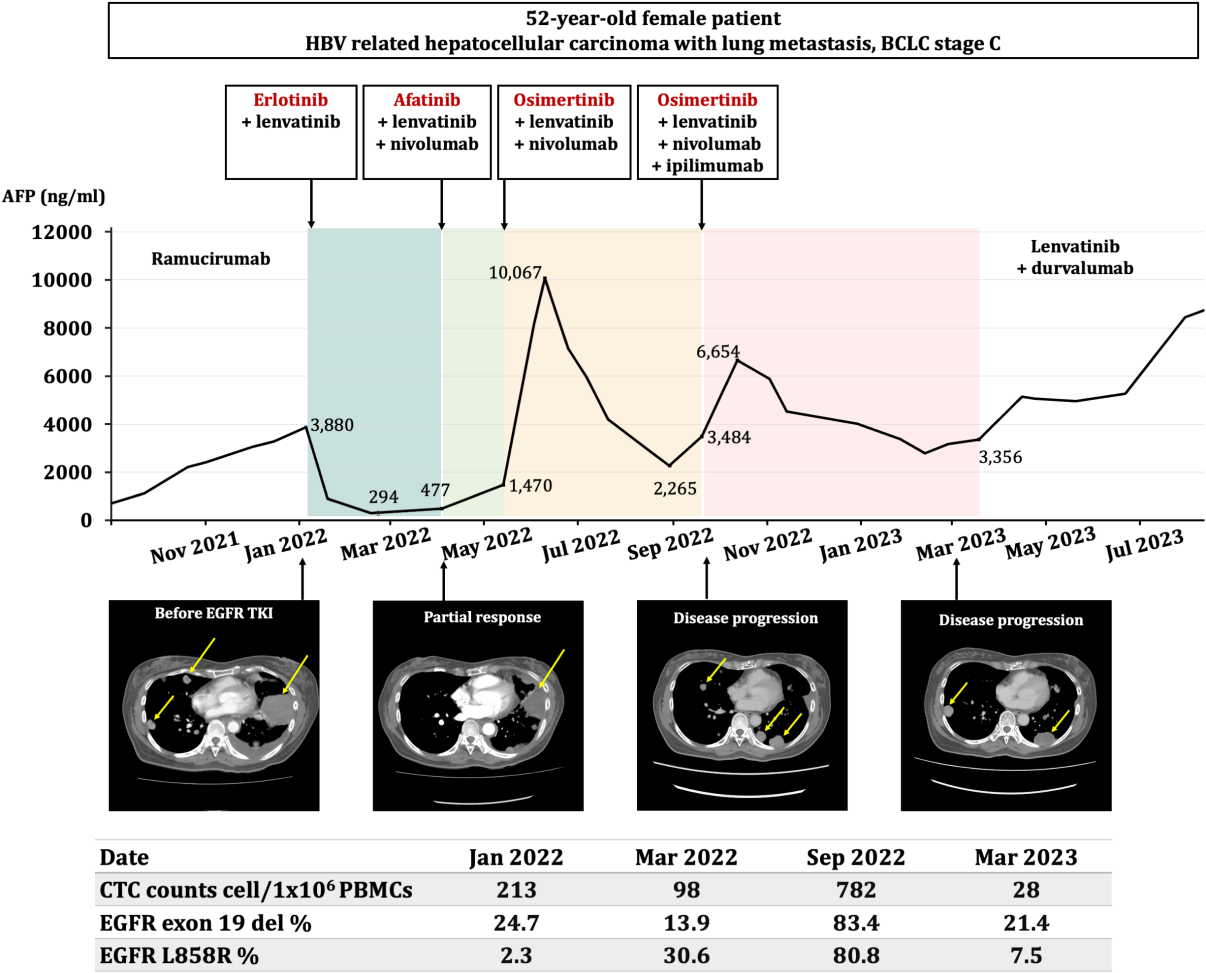
Time course of the present case study. An overview of the treatment regimen, AFP dynamic changes, serial CT of the metastatic lesions and molecular analyses of circulating tumor cells throughout the treatment course is depicted. Text labeled in red, naming erlotinib, afatinib, and osimertinib are EGFR tyrosine kinase inhibitors. Changes of the metastatic lung lesions were highlighted with yellow arrows. HBV, hepatitis B virus; BCLC, Barcelona Clinic Liver Cancer; CTC, circulating tumor cell; PBMC, peripheral blood mononuclear cell; AFP, α-fetoprotein; TKI, tyrosine kinase inhibitor.

Due to an increase in AFP levels from 294 to 477 ng/dl, treatment using erlotinib was switched to afatinib. Anti-PD-1 therapy (nivolumab) was also introduced, alongside continued lenvatinib treatment. However, the patient’s AFP levels continued to increase from 477 to 1,470 ng/dl, which led to the use of osimertinib instead of afatinib, in April 2022. In the following 5 months, there was a notable decline in AFP levels from 10,067-2,265 ng/dl. Unfortunately, in September 2022, the patient’s AFP levels increased from 2,265 to 3,484 ng/dl, accompanied by an increase in CTC count (from 98 to 782 cells/1x10^6^ PBMCs). However, although imaging demonstrated the regression of the previous lesions, several new lesions were reported. Therefore, a four-drug combination therapy consisting of osimertinib (EGFR TKI), lenvatinib (multiple receptor tyrosine kinase), nivolumab (anti-PD-1) and ipilimumab (anti-CTLA-4) was initiated. The tumor remained stable until March 2023 (**Figure 2**). Afterwards, the patient experienced progressive disease and was subsequently treated with lenvatinib and durvalumab. The regimen results in a best response of stable disease with progression-free survival for 6 months. She then received chemotherapy with fluorouracil and oxaliplatin since October 2023 and passed away from terminal HCC in May 2024.

Overall, the patient benefited from EGFR-TKI treatment for 14 months and the treatment was well-tolerated, with only grade 1 dermatosis reported.

## 3 Discussion

The article presented a case in which the patient’s metastatic lung lesions showed a best response of PR to EGFR TKIs, with the treatment’s efficacy lasting for 14 months. In the present case of metastatic HCC, CTCs were detected using an EGFR mutant (exon 19 deletion and L858R) protein. Studies have investigated the association between mutation detection using specific antibodies and the presence of EGFR mutations using quantitative PCR (10-13). For example, a previous study performed immunohistochemistry using anti-EGFR E746-A750 del and anti-EGFR L858R antibodies, which showed high specificity (99.0 and 89.7%, respectively) in non-small cell lung cancer tissue samples. However, their sensitivity (70.6 and 80.4%, respectively) was low (15). The promising association between EGFR immunohistochemical staining and EGFR mutation status has been consistent across numerous studies (16–18).

EGFR upregulation occurs in ~ 66% of HCC cases and has been associated with tumorigenesis, aggressive tumor behavior, metastasis and poor patient survival (19). While EGFR inhibitors such as cetuximab, gefitinib and erlotinib showed therapeutic effect by controlling HCC cell line proliferation and by reducing liver fibrogenesis in HCC animal models (20, 21), objective response rate of these drugs against HCC ranges from 6.6% to 30% (22, 23). Several explanations have been proposed for the limited efficacy in earlier trials: (i) the low prevalence of canonical sensitizing EGFR mutations in HCC (24); (ii) intratumoral heterogeneity and clonal diversity, which limit drug efficacy (25, 26); and (iii) compensatory activation of parallel signaling pathways such as PI3K/AKT and MAPK/ERK (27, 28). These findings suggest that while EGFR is biologically relevant in HCC, classical EGFR inhibitors alone are insufficient to achieve durable responses in unselected populations. More recently, the advance of single-cell sequencing of CTCs has revealed clinically relevant metastatic mechanisms, which would strengthen the rationale for CTC-based molecular profiling as a complementary approach in advanced HCC (29).

Panvichian et al. (30) were among the first to identify both EGFR upregulation and mutations in HCC, and demonstrated upregulation in 32.5% of human HCC tissue samples using immunohistochemistry, as well as identifying a number of mutations in exons 19–23 using sanger sequencing and fragment analysis by capillary electrophoresis (30). It was reported that erlotinib could induce apoptosis and autophagy in cells harboring different EGFR mutations. Furthermore, seven missense EGFR mutations (K757E, N808S, R831C, V897A, P937L, T940A and M947T) were identified in the NIH-3T3 cell line (31).

While studies reporting EGFR mutations in HCC cases are rare, numerous studies have associated the activation of the EGFR pathway with the development of resistance to lenvatinib (32–34). Jin et al. (32) proposed a mechanism where lenvatinib treatment activates the EGFR pathway in liver cancer cells, leading to drug resistance. As a result, it was found that combining erlotinib with lenvatinib effectively silenced MAPK and inhibited HCC cell proliferation. Additionally, combination therapy of gefitinib and lenvatinib reduced tumor volume in both mice and patients with unresectable HCC (trial no. NCT04642547) (21). In summary, the aforementioned studies suggest that EGFR-TKIs may be beneficial for treating HCC, particularly when targeting EGFR pathway activation or mutations.

Genomic analysis of CTCs, obtained through liquid biopsies, is a relatively novel approach for HCC. Using real-time quantitative PCR and Ingenuity Pathway Analysis, Qi et al. (35) identified 67 genes differentially expressed in CTCs that serve roles in cancer cell adhesion, migration, apoptosis and angiogenesis. Among these genes, branched chain amino acid transaminase 1, the most upregulated gene, was associated with chemotherapeutic drug resistance (35). Genetic analysis of CTCs derived from advanced-stage HCC tissues demonstrated the presence of HER2 amplification and p53 deletions (36). Additionally, analysis of CTCs and primary HCC tumor tissues demonstrated concordant somatic copy number alterations (37). Therefore, CTCs offer a valuable alternative to invasive biopsies for analyzing tumor genomics, and potentially pave the way for personalized medicine approaches in HCC.

This study has some limitations that should be noted. While our patient presented with EGFR-mutant circulating tumor cells (CTCs) and showed a favorable response to EGFR TKIs, we were unable to validate this finding with molecular-based EGFR mutation profiling (e.g., polymerase chain reaction or next-generation sequencing) of the tumor biopsy. This decision was guided by several factors. The clinical utility of EGFR mutation profiling in hepatocellular carcinoma (HCC) is challenged by its exceptionally low mutation frequency (~2%) and a high degree of spatial intratumoral heterogeneity (ITH), reported to range from 5.21% to 88.27% (38, 39). Additionally, given the low objective response rate of EGFR TKIs in HCC (6.6–30%) (22, 23), the high cost of such molecular testing is not reimbursed by commercial or National Health Insurance in Taiwan, preventing its adoption as a routine clinical practice. Future research could benefit from integrating comprehensive molecular profiling to better define the genetic characteristics of such cases.

The present report outlines the clinical course and positive outcomes of a patient with metastatic HCC with extensive treatment history, using a precision oncology approach. The present case study suggested that the molecular information provided by CTCs may potentially guide personalized targeted therapy and prolong the survival of patients with advanced HCC.

## Data Availability

The raw data supporting the conclusions of this article will be made available by the authors, without undue reservation.
