# Supplementary material for: Molecular profiling of circulating tumor cells guides effective EGFR inhibitor treatment in advanced hepatocellular carcinoma: a case report
Source: Front Oncol. 2025 Sep 17;15:1608604. doi: 10.3389/fonc.2025.1608604 (PMC12484127; doi:10.3389/fonc.2025.1608604)
Supplement: Supplementary file 1 [file DataSheet1.docx]

Supplementary Material

Supplementary Table 1. Timeline of systemic treatment of presented case

| **Lines** | **Regimens** | **Prescription**  **(dose, frequency)** | **Best response** | **Reason to stop** | **PFS^†^ (months)** | **Period** |
| --- | --- | --- | --- | --- | --- | --- |
| 1L | Bevacizumab  Atezolizumab | 15 mg/kg, Q3W  1200 mg, Q3W | SD^§^ | PD^¶^ | 4 | Aug. 2018 to Dec 2018 |
| 2L | Sorafenib | 400 mg, QD | SD | PD | 5 | Jan 2019 to Jun 2019 |
| 3L | Lenvatinib | 10 mg, QD | SD | PD | 9 | Jul 2019 to Apr 2020 |
| 4L | NBM-BMX-002^%^ | 100 mg, QD | PD | PD | 2 | Apr 2020 to Jun 2020 |
| 5L | Oxaliplatin  Fluorouracil | 85 mg/m^2^, Q2W  2400 mg, Q2W | SD | PD | 5 | Jun 2020 to Nov 2020 |
| 6L | Pembrolizumab  Lenvatinib | 200 mg, Q3W  10 mg, QD | SD | PD | 5 | Dec 2020 to May 2021 |
| 7L | Cabozantinib | 60 mg, QD | SD | PD | 3 | Jun 2021 to Sep 2021 |
| 8L | Ramucirumab | 8 mg/kg, Q2W | PD | PD | 1 | Nov 2021 to Dec 2021 |

^%^NBX-BMX-002 (ClinicalTrials.gov Identifier: NCT03808870) is an orally available new chemical entity to inhibit HDAC8 activity specifically, being developed as a potential anti-cancer therapeutic in Asian subjects with advanced solid tumors by NatureWise.

^†^PFS, progression free survival; ^§^SD, stable disease; ^¶^PD, progressive disease; ^#^PR, partial response.

**Supplementary Table 2. Population percentage of EGFR-mutant CTCs.**

| Time point | Analyzed PBMCs | CTC counts | EGFR^Δ19del^ CTC counts (%) | EGFR^L858R^ CTC counts (%) |
| --- | --- | --- | --- | --- |
| Jan 2022 | 2.5 $\times$ 10^5^ | 213 | 24.70% | 2.30% |
| Mar 2022 | 2.5 $\times$ 10^5^ | 98 | 13.92% | 30.62% |
| Sep 2022 | 2.5 $\times$ 10^5^ | 782 | 83.40% | 80.80% |
| Mar 2023 | 2.5 $\times$ 10^5^ | 28 | 21.40% | 7.50% |


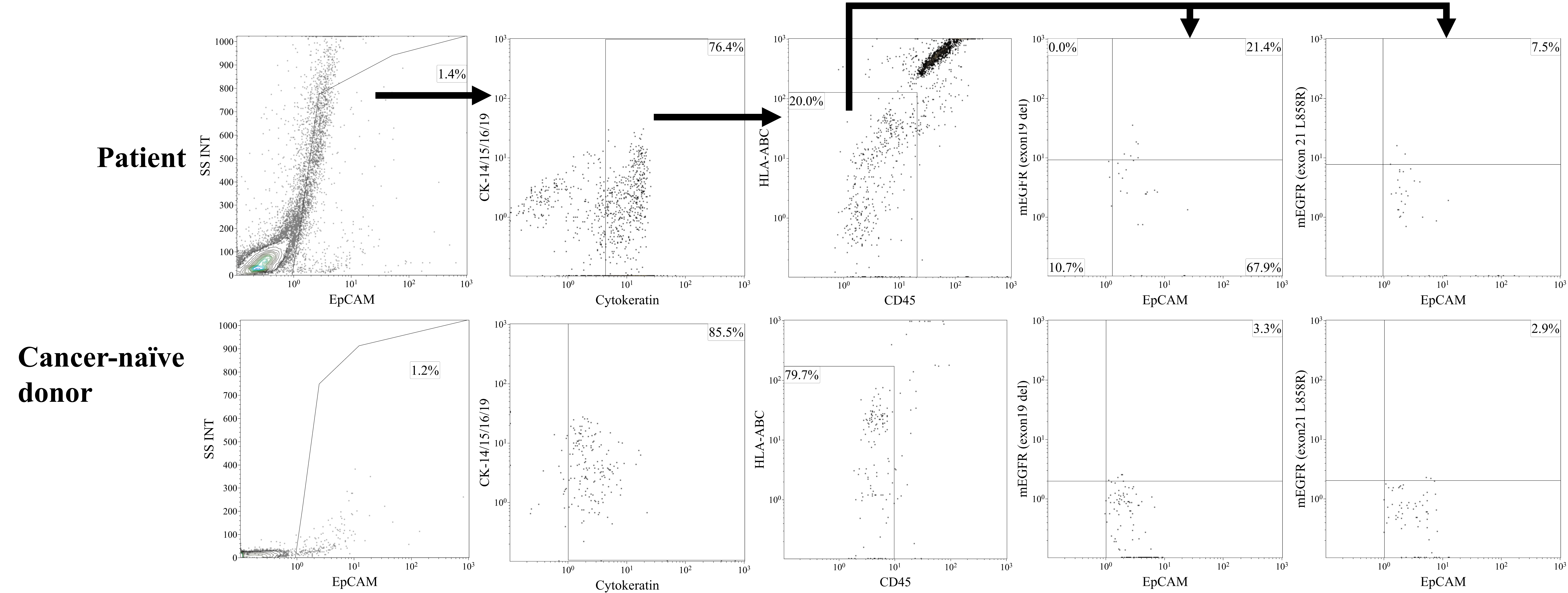


**Supplementary Figure 1. Gating strategy of circulating tumor cells (CTCs) with EGFR mutation**

Fluorescent signals from peripheral blood mononuclear cells were collected using a Gallios flow cytometer. Subsequently, CTCs were identified by a sequential gating strategy with EpCAM^+^, cytokeratin^+^, and CD45^-^HLA-ABC^-^. CTCs with EGFR^Δ19del^ or EGFR^L858R^ mutation were then further identified by gating EpCAM^+^EGFR^Δ19del^ or EpCAM^+^EGFR^L858R^. The gating sequence was indicated with black arrow.


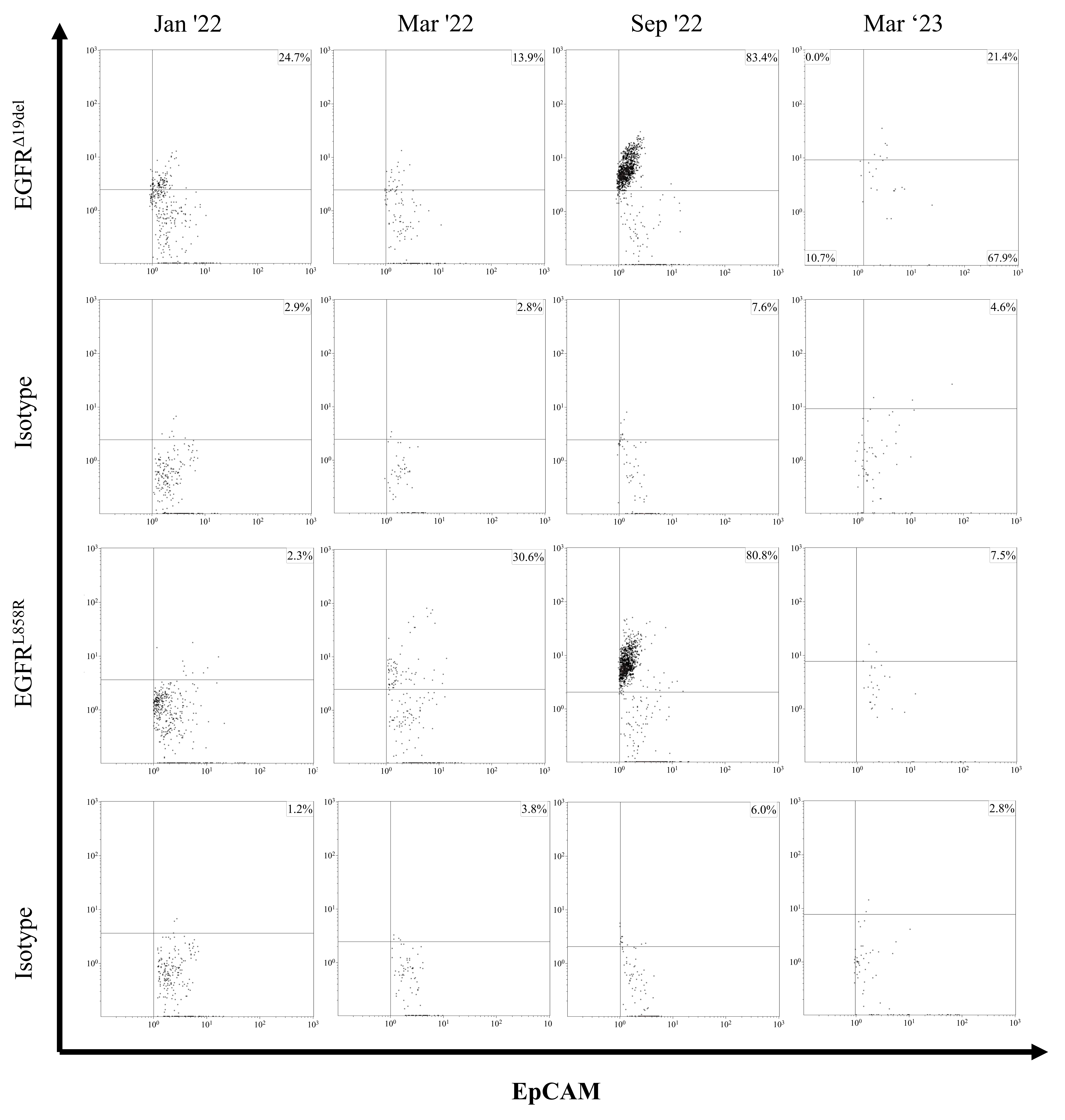


**Supplementary Figure 2. Circulating tumor cells of the patients detected from peripheral blood mononuclear cells at different time points.**
Flow cytometry scatter dot plots showing the percentage of EGFR (E19del)^+^ EpCAM^+^ and EGFR (L858R)^+^ EpCAM^+^ cell subsets in the patient in Jan 2022, March 2022, September 2022, and March 2023. PBMCs from patient and healthy donor were isolated and labeled as described in Case presentation. EpCAM, epithelial cell adhesion molecule.
